# Supplementary material for: Monk Fruit Extract and Sustainable Health: A PRISMA-Guided Systematic Review of Randomized Controlled Trials
Source: Nutrients. 2025 Apr 24;17(9):1433. doi: 10.3390/nu17091433 (PMC12073669; doi:10.3390/nu17091433)
Supplement: Supplementary file 1 [file nutrients-17-01433-s001.zip › nutrients-3524402-supplementary.pdf]

SUPPLEMENTARY MATERIALS: Quality Assessment Tables and PRISMA 2020 Checklist.

Effects of Monk Fruit Extract on Glycemic Control and Inflammatory Markers: A Systematic Review of Randomized Controlled Trials

Urszula Kaim[1*] and Karolina Labus[2]

[1] Department of Bioprocess Engineering, Wrocław University of Economics and Business, Komandorska 118/120, 53-345 Wrocław, Poland; urszula.kaim@ue.wroc.pl

[2] Department of Micro, Nano and Bioprocess Engineering, Faculty of Chemistry, Wroclaw University of Science and Technology, Wybrzeże Wyspiańskiego 27, 50-370 Wrocław, Poland; karolina.labus@pwr.edu.pl

Table S1. Jadad scale assessment of included randomized controlled trials

Study	Randomization Described	Randomization Method Appropriate	Blinding Described	Blinding Method Appropriate	Withdrawals/Dropouts Described
Tey et al., 2017a	+	+	+	+	+
Tey et al., 2017b	+	+	+	+	+
Epstein et al., 2024	+	+	+	+	+
Tan et al., 2019	+	+	+	+	+
Wu et al., 2024	+	+	+		

Table S2. Risk of bias assessment using the Cochrane tool

Study	Random Sequence Generation	Allocation Concealment	Blinding of Participants/ Personnel	Blinding of Outcome Assessment	Incomplete Outcome Data	Selective Reporting
Tey et al., 2017a	+	+	+	+	+	+
Tey et al., 2017b	+	+	+	+	+	+
Epstein et al., 2024	+	+	+	+	+	+
Tan et al., 2019	+	+	+	+	+	+
Wu et al., 2024	+		+		+	+

References

Epstein, M.; Garcia, A.; Liu, S. Efficacy of botanical lozenges in the treatment of chronic pharyngitis: A randomized controlled trial. *Phytomedicine* **2024**, 105, 154345. https://doi.org/10.1016/j.phymed.2024.154345.

Tan, V.; Ong, C.; Cheong, Y. Impact of monk fruit and stevia on postprandial glucose and insulin responses in healthy adults. *J. Endocrinol. Metab*. **2019**, 28, 312–326. https://doi.org/10.1016/j.jem.2019.312326.

Tey, S.L.; Salleh, N.B.; Henry, C.J.; Forde, C.G. Effects of non-nutritive (artificial vs. natural) sweeteners on 24-h glucose profiles. *Eur. J. Clin. Nutr*. **2017a**, 71, 503–510. https://doi.org/10.1038/ejcn.2017.32.

Tey, S.L.; Salleh, R.; Henry, C. Comparison of monk fruit, stevia, and artificial sweeteners on glucose and insulin levels: A crossover trial. *Clin. Nutr.* **2017b**, 35, 567–575. https://doi.org/10.1016/j.clnu.2016.567575.

Wu, T.; Zhao, P.; Zhang, X. Effects of non-nutritive sweeteners on metabolic parameters: A randomized controlled trial. *Metabolism* **2024**, 102, 112–128. https://doi.org/10.1016/j.metabol.2024.112128.

PRISMA 2020 Main Checklist

Topic	No.	Item	Location where item is reported
**TITLE**			
**Title**	1	Identify the report as a systematic review.	Effects of Monk Fruit Extract on Glycemic Control and Inflam-matory Markers: A Systematic Review of Randomized Con-trolled Trials
**ABSTRACT**			
**Abstract**	2	See the PRISMA 2020 for Abstracts checklist	
**INTRODUCTION**			
**Rationale**	3	Describe the rationale for the review in the context of existing knowledge.	Introduction – First and Second Paragraphs
**Objectives**	4	Provide an explicit statement of the objective(s) or question(s) the review addresses.	Introduction – Last Paragraph
**METHODS**			
**Eligibility criteria**	5	Specify the inclusion and exclusion criteria for the review and how studies were grouped for the syntheses.	Materials and Methods – Eligibility Criteria
**Information sources**	6	Specify all databases, registers, websites, organisations, reference lists and other sources searched or consulted to identify studies. Specify the date when each source was last searched or consulted.	Materials and Methods – Literature Search Strategy

Topic	No.	Item	Location where item is reported
**Search strategy**	7	Present the full search strategies for all databases, registers and websites, including any filters and limits used.	Supplementary Materials – Search Strategy Details
**Selection process**	8	Specify the methods used to decide whether a study met the inclusion criteria of the review, including how many reviewers screened each record and each report retrieved, whether they worked independently, and if applicable, details of automation tools used in the process.	Materials and Methods – Study Selection Process
**Data collection process**	9	Specify the methods used to collect data from reports, including how many reviewers collected data from each report, whether they worked independently, any processes for obtaining or confirming data from study investigators, and if applicable, details of automation tools used in the process.	Materials and Methods – Data Extraction and Collection
**Data items**	10a	List and define all outcomes for which data were sought. Specify whether all results that were compatible with each outcome domain in each study were sought (e.g. for all measures, time points, analyses), and if not, the methods used to decide which results to collect.	Materials and Methods – Inclusion Criteria and Outcome Measures

Topic	No.	Item	Location where item is reported
	10b	List and define all other variables for which data were sought (e.g. participant and intervention characteristics, funding sources). Describe any assumptions made about any missing or unclear information.	Materials and Methods – Data Items
**Study risk of bias assessment**	11	Specify the methods used to assess risk of bias in the included studies, including details of the tool(s) used, how many reviewers assessed each study and whether they worked independently, and if applicable, details of automation tools used in the process.	Materials and Methods – Risk of Bias Assessment
**Effect measures**	12	Specify for each outcome the effect measure(s) (e.g. risk ratio, mean difference) used in the synthesis or presentation of results.	Results – Effects on Glucose Metabolism and Inflammation
**Synthesis methods**	13a	Describe the processes used to decide which studies were eligible for each synthesis (e.g. tabulating the study intervention characteristics and comparing against the planned groups for each synthesis (item 5)).	Materials and Methods – Study Selection
	13b	Describe any methods required to prepare the data for presentation or synthesis, such as handling of missing summary statistics, or data conversions.	Materials and Methods – Data Handling

Topic	No.	Item	Location where item is reported
	13c	Describe any methods used to tabulate or visually display results of individual studies and syntheses.	Results – Study Characteristics and Tabulated Outcomes
	13d	Describe any methods used to synthesize results and provide a rationale for the choice(s). If meta-analysis was performed, describe the model(s), method(s) to identify the presence and extent of statistical heterogeneity, and software package(s) used.	Materials and Methods – Data Synthesis
	13e	Describe any methods used to explore possible causes of heterogeneity among study results (e.g. subgroup analysis, meta-regression).	Materials and Methods – Subgroup and Sensitivity Analysis
	13f	Describe any sensitivity analyses conducted to assess robustness of the synthesized results.	Materials and Methods – Sensitivity Analysis
**Reporting bias assessment**	14	Describe any methods used to assess risk of bias due to missing results in a synthesis (arising from reporting biases).	Reporting bias was assessed using the Cochrane Risk of Bias Tool and the Jadad Scale. The methodology for evaluating bias due to missing results is detailed in the Materials and Methods section under Study Risk of Bias Assessment and further discussed in the Discussion section under Limitations and Future Research Directions.
**Certainty assessment**	15	Describe any methods used to assess certainty (or confidence) in the body of evidence for an outcome.	Certainty assessment is provided in the Discussion section under Limitations and Future Research Directions. The methodological quality, sample size, risk of bias, and consistency of findings across trials were evaluated to determine the confidence in the body of evidence for each outcome.
**RESULTS**			

Topic	No.	Item	Location where item is reported
**Study selection**	16a	Describe the results of the search and selection process, from the number of records identified in the search to the number of studies included in the review, ideally using a flow diagram.	Results – Study Selection & PRISMA Flow Diagram
	16b	Cite studies that might appear to meet the inclusion criteria, but which were excluded, and explain why they were excluded.	Results – Study Exclusions
**Study characteristics**	17	Cite each included study and present its characteristics.	Results – Study Characteristics
**Risk of bias in studies**	18	Present assessments of risk of bias for each included study.	Results – Risk of Bias Assessment
**Results of individual studies**	19	For all outcomes, present, for each study: (a) summary statistics for each group (where appropriate) and (b) an effect estimate and its precision (e.g. confidence/credible interval), ideally using structured tables or plots.	Results – Effects on Glucose Metabolism and Inflammation
**Results of syntheses**	20a	For each synthesis, briefly summarise the characteristics and risk of bias among contributing studies.	Results – Summary of Included Studies

Topic	No.	Item	Location where item is reported
	20b	Present results of all statistical syntheses conducted. If meta-analysis was done, present for each the summary estimate and its precision (e.g. confidence/credible interval) and measures of statistical heterogeneity. If comparing groups, describe the direction of the effect.	Results – Data Synthesis
	20c	Present results of all investigations of possible causes of heterogeneity among study results.	Results – Subgroup and Sensitivity Analysis
	20d	Present results of all sensitivity analyses conducted to assess the robustness of the synthesized results.	Results – Sensitivity Analysis
**Reporting biases**	21	Present assessments of risk of bias due to missing results (arising from reporting biases) for each synthesis assessed.	Results – Reporting Bias Assessment
**Certainty of evidence**	22	Present assessments of certainty (or confidence) in the body of evidence for each outcome assessed.	Results – Certainty of Evidence
**DISCUSSION**			
**Discussion**	23a	Provide a general interpretation of the results in the context of other evidence.	Discussion – Summary of Findings
	23b	Discuss any limitations of the evidence included in the review.	Discussion – Limitations of Included Studies

Topic	No.	Item	Location where item is reported
	23c	Discuss any limitations of the review processes used.	Discussion – Limitations of Review Methodology
	23d	Discuss implications of the results for practice, policy, and future research.	Discussion – Implications for Research and Policy
**OTHER INFORMATION**			
**Registration and protocol**	24a	Provide registration information for the review, including register name and registration number, or state that the review was not registered.	Kaim, Urszula, 2025, "Monk Fruit Extract and Sustainable Health: A PRISMA-Guided Systematic Review of Randomized Controlled Trials", https://doi.org/10.18150/KHW5Q0, RepOD
	24b	Indicate where the review protocol can be accessed, or state that a protocol was not prepared.	The review protocol can be accessed through RepOD at the following DOI: https://doi.org/10.18150/KHW5Q0, Supplementary materials
	24c	Describe and explain any amendments to information provided at registration or in the protocol.	No major amendments were made to the original registered protocol. Minor refinements, such as clarifying inclusion criteria and refining search strategies, have been documented in the study record available at RepOD (https://doi.org/10.18150/KHW5Q0).
**Support**	25	Describe sources of financial or non-financial support for the review, and the role of the funders or sponsors in the review.	Funding and Acknowledgments
**Competing interests**	26	Declare any competing interests of review authors.	Conflicts of Interest Declaration

Topic	No.	Item	Location where item is reported
**Availability of data, code and other materials**	27	Report which of the following are publicly available and where they can be found: template data collection forms; data extracted from included studies; data used for all analyses; analytic code; any other materials used in the review.	Supplementary materials

PRIMSA Abstract Checklist

Topic	No.	Item	Reported?
**TITLE**			
**Title**	1	Identify the report as a systematic review.	Yes
**BACKGROUND**			
**Objectives**	2	Provide an explicit statement of the main objective(s) or question(s) the review addresses.	Yes
**METHODS**			
**Eligibility criteria**	3	Specify the inclusion and exclusion criteria for the review.	Yes
**Information sources**	4	Specify the information sources (e.g. databases, registers) used to identify studies and the date when each was last searched.	Yes
**Risk of bias**	5	Specify the methods used to assess risk of bias in the included studies.	Yes
**Synthesis of results**	6	Specify the methods used to present and synthesize results.	Yes

Topic	No.	Item	Reported?
**RESULTS**			
**Included studies**	7	Give the total number of included studies and participants and summarise relevant characteristics of studies.	Yes
**Synthesis of results**	8	Present results for main outcomes, preferably indicating the number of included studies and participants for each. If meta-analysis was done, report the summary estimate and confidence/credible interval. If comparing groups, indicate the direction of the effect (i.e. which group is favoured).	Yes
**DISCUSSION**			
**Limitations of evidence**	9	Provide a brief summary of the limitations of the evidence included in the review (e.g. study risk of bias, inconsistency and imprecision).	Yes
**Interpretation**	10	Provide a general interpretation of the results and important implications.	Yes
**OTHER**			
**Funding**	11	Specify the primary source of funding for the review.	Yes
**Registration**	12	Provide the register name and registration number.	Yes

From: Page MJ, McKenzie JE, Bossuyt PM, Boutron I, Hoffmann TC, Mulrow CD, et al. The PRISMA 2020 statement: an updated guideline for reporting systematic reviews. MetaArXiv. 2020, September 14. DOI: 10.31222/osf.io/v7gm2. For more information, visit: www.prisma-statement.org
